# Potential application of traditional Chinese medicine in age-related macular degeneration—focusing on mitophagy

**DOI:** 10.3389/fphar.2024.1410998

**Published:** 2024-05-17

**Authors:** Yujia Yu, Gaofeng Wang, Yong Liu, Zhaoru Meng

**Affiliations:** ^1^ First Clinical Medical School, Shandong University of Traditional Chinese Medicine, Jinan, Shandong, China; ^2^ Affiliated Hospital of Shandong University of Traditional Chinese Medicine, Shandong Province Hospital of Traditional Chinese Medicine, Jinan, Shandong, China; ^3^ School of Engineering, The Hong Kong University of Science and Technology, Kowloon, Hong Kong SAR, China

**Keywords:** age-related macular degeneration, mitophagy, oxidative stress, traditional Chinese medicine, mechanism

## Abstract

Retinal pigment epithelial cell and neuroretinal damage in age-related macular degeneration (AMD) can lead to serious visual impairments and blindness. Studies have shown that mitophagy, a highly specialized cellular degradation system, is implicated in the pathogenesis of AMD. Mitophagy selectively eliminates impaired or non-functioning mitochondria via several pathways, such as the phosphatase and tensin homolog-induced kinase 1/Parkin, BCL2-interacting protein 3 and NIP3-like protein X, FUN14 domain-containing 1, and AMP-activated protein kinase pathways. This has a major impact on the maintenance of mitochondrial homeostasis. Therefore, the regulation of mitophagy could be a promising therapeutic strategy for AMD. Traditional Chinese medicine (TCM) uses natural products that could potentially prevent and treat various diseases, such as AMD. This review aims to summarize recent findings on mitophagy regulation pathways and the latest progress in AMD treatment targeting mitophagy, emphasizing methods involving TCM.

## 1 Introduction

**FIGURE 1:**
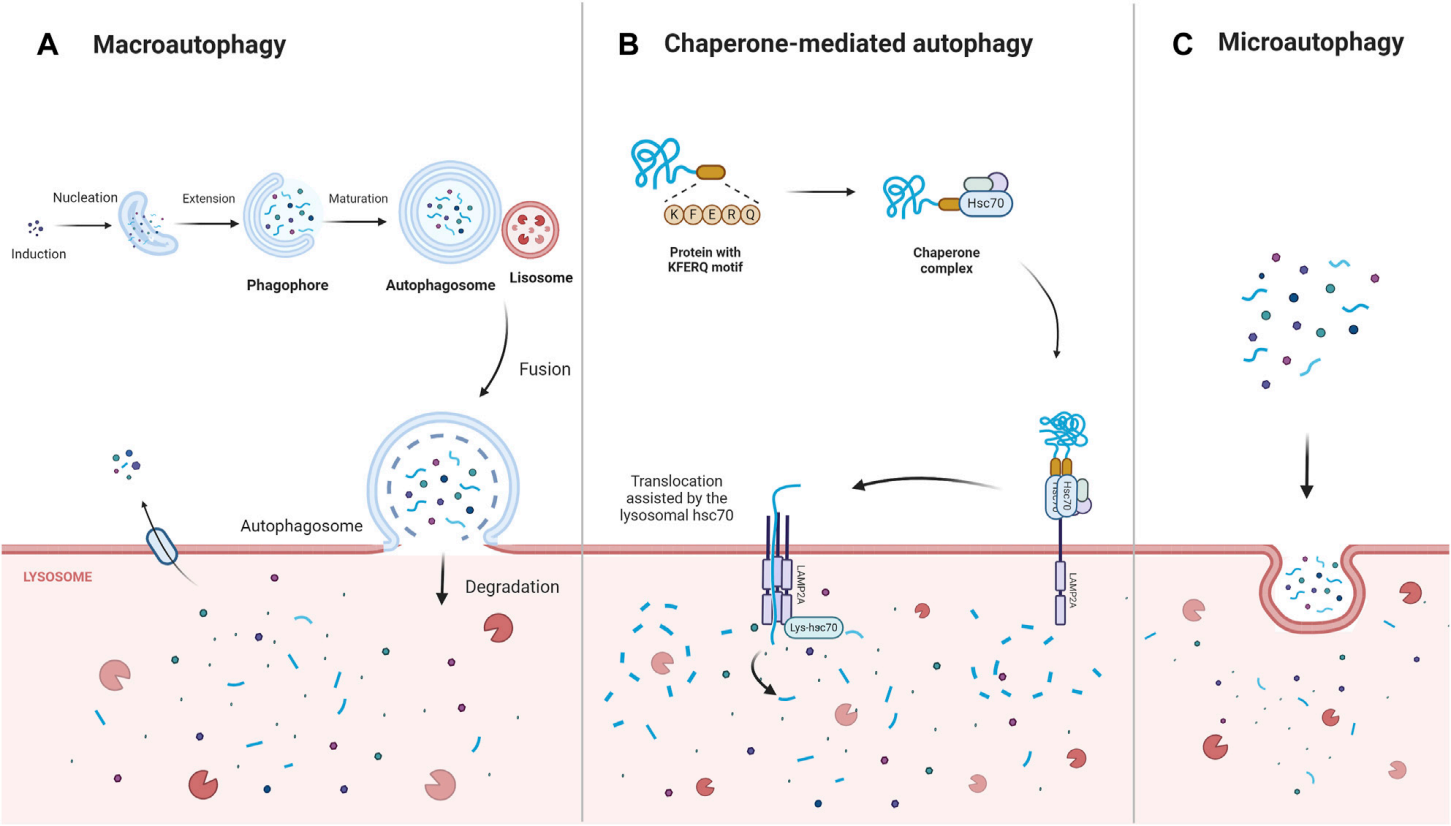
The main types of autophagy: macroautophagy, chaperone- mediated autophagy, microautophagy. Macroautophagy is induced, nucleated, and extended to form a double-membrane structure autophagosome, which wraps around the degraded material and ultimately fuses with the lysosome for degradation. Chaperone-mediated autophagy directly recognizes proteins with KFERQ-related motifs by means of the chaperone protein Hsc70, and the receptor protein LAMP2A on the lysosomal membrane recognizes the KFERQ motifs exposed by the binding protein and guides the target protein into the lysosome for degradation. In contrast, microautophagy directly engulfs the degraded material mainly through the invagination of the lysosomal membrane. Abbreviations: Lysosomal-associated membrane protein 2A (LAMP2A).

Autophagy is a multifunctional degradation system that helps cells maintain homeostasis by encapsulating cytoplasmic proteins, damaged organelles, and pathogens into vesicles (autophagosomes), which subsequently fuse with lysosomes to create autolysosomes, leading to the degradation of the encapsulated cargo and the generation of amino acids, nucleotides, sugars, fatty acids, and adenosine triphosphate (ATP). By assisting in the regulation of protein, nucleic acid, and lipid balances, the modulation of reactive oxidative stress and oxygen species (ROS), and the improvement of mitochondrial function, autophagy contributes to cellular metabolic needs and the renewal of intracellular organelles (Nita and Grzybowski, 2023). Depending on the intracellular lysosomal degradation mechanism, three distinct forms of autophagy—macroautophagy, microautophagy, and chaperone-mediated autophagy—have been distinguished (Mizushima, 2018; Yao and Shen, 2020) (Figure 1). Macroautophagy is the most predominant and conserved type of autophagy and proceeds via five stages—induction, nucleation, extension, fusion, and degradation (Nieto-Torres and Hansen, 2021). During chaperone-mediated autophagy, the chaperone Hsc70 and co-chaperones identify target proteins containing KFERQ-like motifs and then transport these proteins to the lysosomal membrane to bind to the lysosomal receptor lysosomal-associated membrane protein 2A (LAMP2A), triggering receptor multimerization, cargo internalization, and degradation (Gómez-Sintes and Arias, 2021). In contrast, during microautophagy, the target proteins are directly engulfed by the lysosomal membrane through invagination (Fleming et al., 2022).

Mitophagy, a highly specialized type of autophagy, regulates mitochondrial fission and fusion, thereby eliminating malfunctioning and impaired mitochondria, promoting mitochondrial renewal, preventing ROS overproduction, inhibiting potential cellular oxidative damage, and ensuring mitochondrial quality (Wen et al., 2023). In response to ROS generation, nutrient deficiency, cellular senescence, and other factors, intracellular mitochondria are damaged and depolarized. During mitophagy, the impaired mitochondria are incorporated into autophagosomes, which then fuse with lysosomes, resulting in the breakdown of these organelles (Lu et al., 2023). Mitophagy is also involved in numerous physiological functions, such as delaying the ageing process and cell differentiation. Interference in these processes induces physiological senescence and several age-related diseases (Krantz et al., 2021; Banarase et al., 2023; Sanz et al., 2023). Recent evidence indicates that impaired mitochondrial energy metabolism and age-related diseases share pathological features, such as an increased mutation rate in mitochondrial DNA (mtDNA), impaired electron transport chain function, elevated ROS levels, and the enhanced release of pro-apoptotic factors (Fang et al., 2017). As a result, the dysregulation of mitophagy can induce mitochondrial dysfunction, leading to the progression of age-related diseases.

**FIGURE 2:**
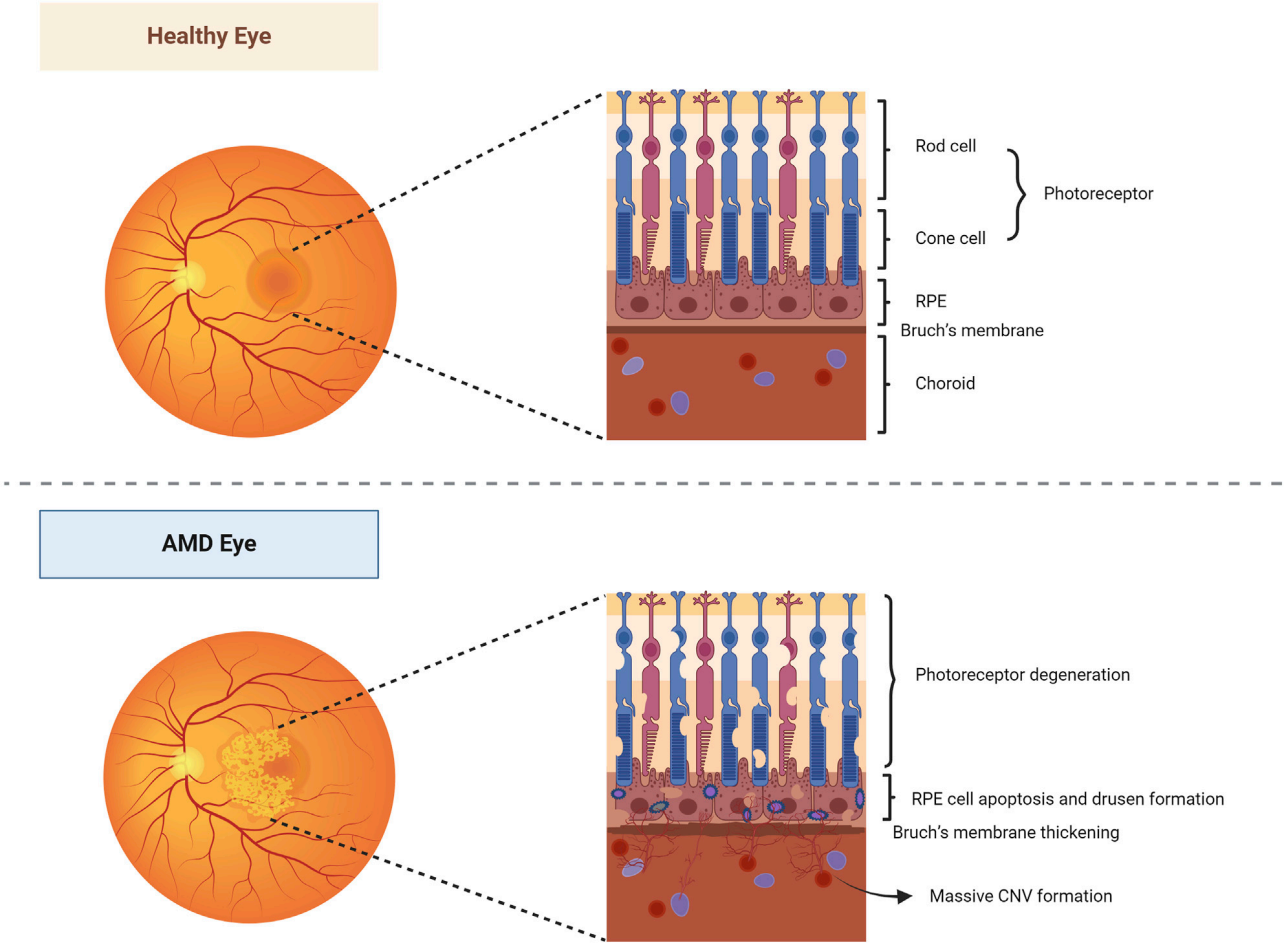
Healthy and AMD-affected eyes. In healthy eyes, the fundus is normal, and the morphology and structure of the photoreceptor cells, RPE layer, Bruch’s membrane, and choroidal layer in the macula are normal. In AMD eyes, the pigmentation of the macular area was disorganized, and a slightly elevated neovascular membrane could be seen locally. Its photoreceptor cells, RPE, Bruch’s membrane, and choroidal multilayer organization changed constantly, resulting in degeneration of photoreceptor cells, apoptosis of RPE cells, pigmentation production, thickening of Bruch’s membrane, and formation of a large number of CNV. Abbreviations: AMD, age-related macular degeneration; RPE, retinal pigment epithelium; CNV, choroidal neovascularization.

Age-related macular degeneration (AMD) is a lesion on the macula that primarily affects the retinal pigment epithelium (RPE), photoreceptor cells, Bruch’s membrane, and choroidal multilayered tissue. Gradual degeneration of the outer retina and the formation of new blood vessels between the retina and Bruch’s membrane can advance to geographic atrophic or choroidal neovascular AMD, commonly known as dry and wet AMD, respectively (Figure 2). It is estimated that the worldwide occurrence of AMD stands at 196 million, with projections suggesting an increase to 288 million by 2040 (Xiao et al., 2023). RPE cells are known for high oxygen consumption, continuous photostimulation, and susceptibility to lipid peroxidation product exposure (Kaarniranta et al., 2009). Consequently, oxidative stress and damage play a crucial role in the progression of RPE degeneration in AMD (Handa, 2012; Blasiak et al., 2013). Persistent oxidative stress on RPE cells may result in the accumulation of extracellular deposits and damaged organelles, nucleic acids, lipids, cellular proteins, and lipofuscin granules, thereby increasing the levels of ROS. These excessive levels of oxidized lipoproteins and ROS induce protein misfolding, aggregation, and persistent activation of the innate immune responses, leading to protein accumulation, mitochondrial dysfunction, and inflammasome activation in the RPE cells (Ferrington et al., 2016; Kauppinen et al., 2016; Piippo et al., 2018; Kaarniranta et al., 2019a; Kaarniranta et al., 2020). Mitochondria are the important sites of retinal oxidation and are mainly found within the RPE. Mitochondrial dysfunction reduces oxidative phosphorylation, results in excessive ROS production, increases mtDNA damage and mutations, and enhances the release of pro-inflammatory and pro-apoptotic factors, which induce oxidative stress, inflammatory response, and apoptosis (Murphy, 2009; Kageyama et al., 2012; Kaarniranta et al., 2020). Studies have shown that mitophagy regulates RPE cell damage and apoptosis by maintaining mitochondrial function and protein folding (Dhirachaikulpanich et al., 2022; Hyttinen et al., 2023).

Meanwhile, excessive accumulation of extracellular deposits inhibits mitophagy and promotes inflammation in AMD, whereas reduced accumulation restores mitophagy in RPE cells, alleviating AMD (Yu et al., 2023). Damage to mtDNA might be crucial in advancing AMD (Kaarniranta et al., 2019b). Recent reports have indicated that mtDNA damage increases with age; furthermore, elevated mtDNA damage, a higher number of mutations, and reduced DNA repair efficacy have been reported to be associated with the onset and staging of AMD (Piippo et al., 2018). Feher et al. (2006) reported a notable decline in the number of mitochondria and the absence of stromal density and cristae in the RPE of individuals with AMD compared to those in the control group. Zhao et al. (2011) stated that the suppression of oxidative phosphorylation in mouse RPE mitochondria led to the activation of the mechanistic target of the rapamycin (mTOR) pathway in an AMD mouse model. Proteomic analyses performed by Nordgaard et al. (2006) showed the differential expression of mitochondrial refolding and trafficking-related proteins in the RPE of AMD patients compared to that in the non-AMD controls. Subsequent research (Nordgaard et al., 2008) demonstrated that depending on the stage of AMD, the expression of mtHsp70, the mitochondrial translation factor Tu, mitofilin, subunit VIb of the cytochrome c oxidase complex, and α-, β-, and δ-subunits of the catalytic portion of ATP synthase was affected in the RPE. Using an *in vitro* model of AMD generated by fusing mitochondria-rich platelets from patients with AMD and mitochondrially-depleted retinal pigment epithelial cells (ARPE-19), Dohl et al. (2022) reported increased concentrations of ROS, which could result in mtDNA damage, enhanced antioxidant responses, and increased expression levels of anti-inflammatory proteins. These results suggested that mtDNA damage response was fundamental in preventing AMD and slowing its progression. New findings indicate that boosting autophagy in RPE cells could be an innovative approach to tackle AMD.

Nevertheless, there are few approved mitophagy-modulating medications for AMD treatment. Current clinical AMD therapy includes lifestyle improvements (e.g., caloric restriction and moderate exercise) as well as pharmacological treatments [e.g., antioxidants (D Aloisio et al., 2022), tyrosine kinase inhibitors (Das et al., 2023), antidiabetic drugs (Thee et al., 2021; Mauschitz et al., 2022), anti-vascular endothelial growth factor (anti-VEGF), and other therapies (Francisco and Rowan, 2023; Servillo et al., 2023; Szigiato et al., 2023)]. Lately, traditional Chinese medicine (TCM) has garnered increasing attention for its antioxidant, anti-apoptotic, anti-inflammatory, and lipid-reducing activities, positioning it as a promising treatment option for AMD (Cao et al., 2022a; Yu et al., 2024). This review focuses on the molecular mechanisms governing mitophagy and innovative treatment approaches for employing TCM as a potential AMD therapy.

## 2 Mitophagy-specific pathways in AMD

### 2.1 Mitophagy mediated by PINK1/Parkin

**FIGURE 3:**
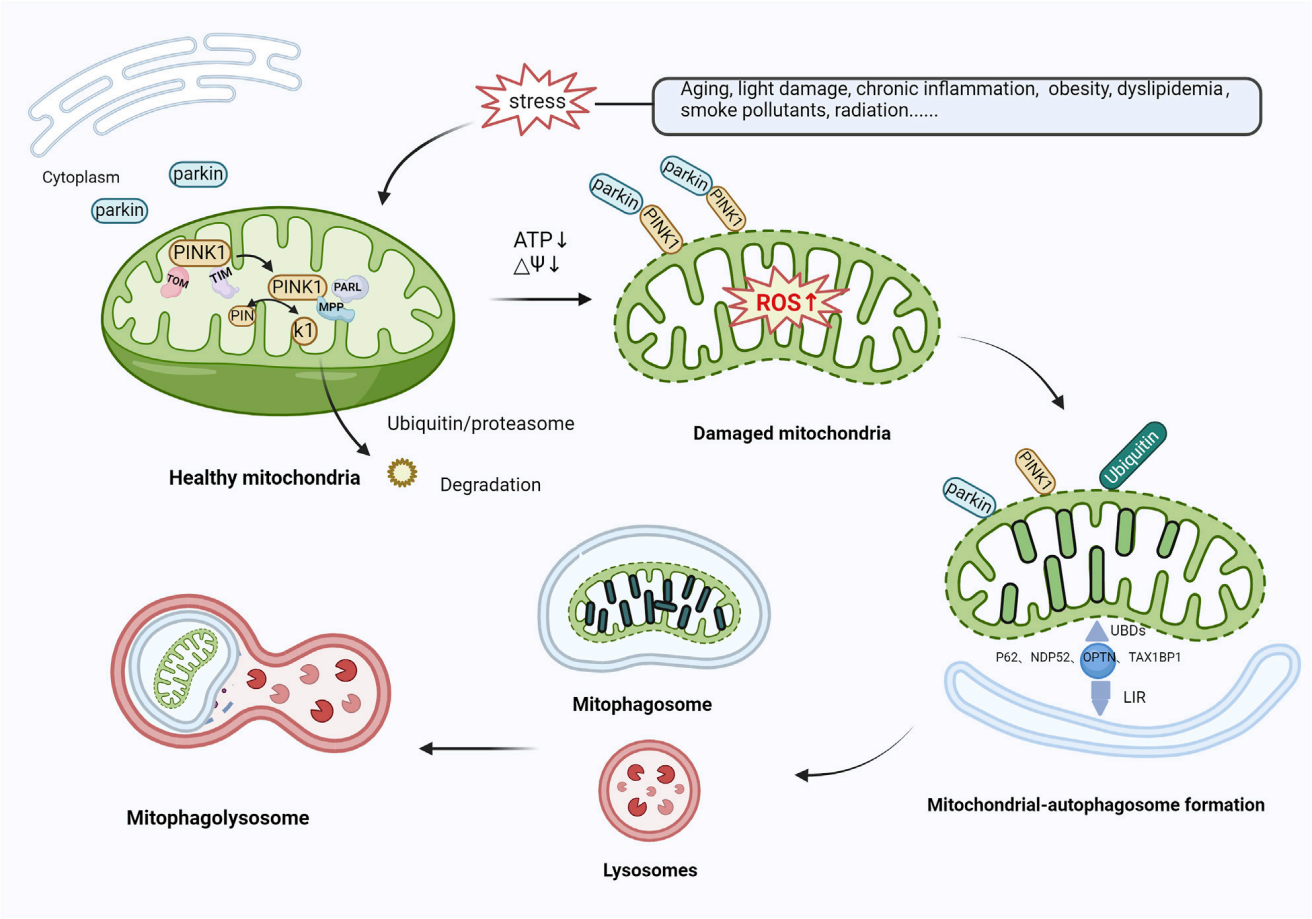
Mitophagy activation mediated by PINK1/Parkin. In healthy mitochondria, PINK1 is transported to mitochondria via TOM and TIM, cleaved by PARL and MPP, and then translocated to the cytoplasm, where it is broken down by the ubiquitin/proteasomal pathway. In response to various stressors, the level of ROS production rises, inducing oxidative damage. PINK1 recognizes damaged mitochondria, accumulates in large quantities in the mitochondrial outer membrane, and recruits and phosphorylates Parkin, activating its E3 ubiquitin ligase function. Both proteins translocate from the cytoplasm to the mitochondrial outer membrane and ubiquitinate mitochondrial proteins by forming polyubiquitin chains. Autophagy-related proteins (e.g., P62, NDP52, OPTN, TAX1BP1) then recognize ubiquitinated mitochondria through UBDs on the one hand, and on the other hand, are anchored to the membrane of autophagic vesicles through the LIR to form mitochondrial autophagosomes, which subsequently merge with lysosomes, leading to mitochondrial degradation. Abbreviations: PINK1, phosphatase and tensin homolog (PTEN)-induced kinase 1; TOM, translocase of outer membrane; TIM, Translocase of inner membrane; PARL, presenilin-associated rhomboid-like; MPP, mitochondrial-processing protease; ROS, reactive oxygen species; NDP52, nuclear dot protein 52; OPTN, optineurin; TAX1BP1, TAX1‐binding protein‐1; UBDs, Ubiquitin-binding domains; LIR, LC3-interacting region.

Various mechanisms linked to mitophagy, which play a role in the onset of AMD, have been identified. The PTEN-induced putative kinase protein 1 (PINK1)/Parkin-mediated ubiquitination degradation pathway is the most characterized ubiquitin-dependent signaling pathway responsible for the control of mitochondrial structure and function (Bowling et al., 2019). Under normal conditions, PINK1 and Parkin are present at low levels in the mitochondrial outer membrane and cytoplasm, respectively. The cytoplasmic synthesis of PINK1 is followed by its relocation to the mitochondria, which is facilitated by the interaction between translocase of the outer membrane (TOM) and translocase of the inner membrane (TIM) (Kato et al., 2013; Sekine et al., 2019). Subsequently, PINK1 is cleaved by presenilin-associated rhomboid-like (PARL) (Meissner et al., 2015) and mitochondrial-processing protease (MPP) (Bayne and Trempe, 2019), leading to the degradation of PINK1 via the ubiquitin/proteasome pathway (Yamano and Youle, 2013). In response to stress, mitochondria are depolarized, and the level of ROS production increases, thereby inducing oxidative damage. During mitochondrial dysfunction, PINK1 degradation is blocked, causing accumulation at the mitochondrial outer membrane, the recruitment and phosphorylation of Parkin, and the activation of E3 ubiquitin ligase function. Subsequently, PINK1 and Parkin translocate from the cytoplasm to the mitochondrial outer membrane and ubiquitinate mitochondrial component proteins by forming polyubiquitin chains (Sauvé et al., 2022). Autophagy-associated proteins, such as nuclear dot protein 52 (NDP52), TAX1‐binding protein‐1 (TAX1BP1), optineurin (OPTN), and p62 (Shang et al., 2019; Zachari et al., 2020; Nguyen et al., 2023), recognize ubiquitinated mitochondria through their ubiquitin-binding domains (UBDs) and anchor their cargo to autophagic vesicle membranes via their LC3-interacting region (LIR) motifs, contributing to the formation of mitochondrial autophagosomes. Ultimately, autophagosomes fuse with lysosomes to form mature mitochondrial autolysosomes and trigger mitochondrial degradation (Callegari et al., 2017; Urbina-Varela et al., 2020) (Figure 3). Datta et al. (2023) reported that PINK1 levels were reduced in centro-concave RPE cells obtained from individuals with early AMD. Oxidative stress causes an increase in the concentration of heat shock protein Hsp70 in the lysosomes of RPE cells from patients with AMD, inhibiting the build-up of cytotoxic protein aggregates, alleviating lipofuscin-induced misfolding of intracellular proteins, and activating autophagy-mediated protein hydrolysis, thereby protecting RPE cells from oxidative stress (Kaarniranta et al., 2009; Subrizi et al., 2015). Hsp70 functions as a PINK1 degradation regulator, while Hsp70-interacting proteins, BCL2-associated athanogene 5 (BAG5) and BCL2-associated athanogene 2 (BAG2), play a significant role in regulating PINK1 stability by reducing its ubiquitination. Lowering Hsp70 levels downregulates PINK/Parkin-mediated mitophagy (Zheng et al., 2018a). Accordingly, the mechanism of Hsp70 action that leads to the protection of RPE cells via PINK1-mediated mitophagy could be significant in AMD progression. The regulation of antioxidant production and mitochondrial biosynthesis relies heavily on the involvement of peroxisome proliferator-activated receptor gamma coactivator 1-alpha (PGC-1α) and nuclear factor erythroid 2-related factor 2 (NFE2L2), respectively. These proteins upregulate antioxidant parameters and prevent the mitochondrial injury and apoptosis caused by ROS (Kaarniranta et al., 2020). Within the confines of the retina of NFE2L2/PGC-1α double knockout mice, a dry AMD model, high oxidative stress levels, protein aggregation, the significant upregulation of PINK1/Parkin expression, impaired mitochondrial injury, reduced mitophagy, and aberrant autophagic flux in RPE cells were observed. At the same time, the control group displayed minimal or no PINK1/Parkin activity (Sridevi Gurubaran et al., 2020). These results further demonstrate that controlling PINK1/Parkin-mediated mitophagy could be a viable approach to treating AMD.

### 2.2 Mitophagy mediated by BNIP3/NIX

**FIGURE 4:**
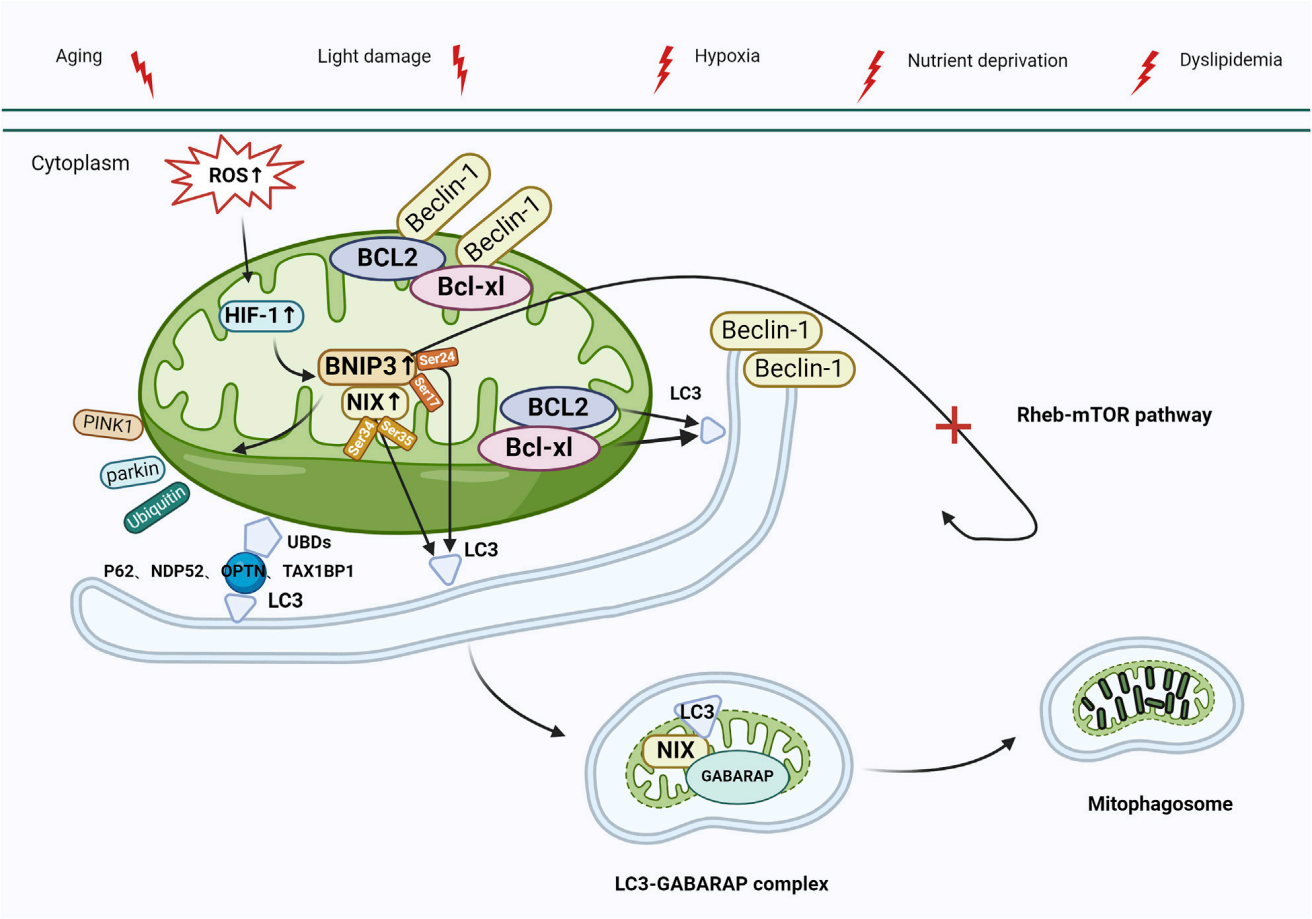
Mitophagy activation mediated by BNIP3/NIX. Under stress (e.g., aging, light damage, hypoxia, nutrient deprivation, and dyslipidemia), elevated levels of ROS activate HIF-1, leading to the upregulation of BNIP3/NIX, which interacts with the Beclin-1/BCL-xl and Beclin-1/BCL2 complexes, releasing Beclin-1, which activates autophagy. BNIP3/NIX-mediated mitophagy can also be regulated by phosphorylation. Ser17 and Ser24 phosphorylated on BNIP3 bind to LC3 via LIR, whereas Ser34 and Ser35 phosphorylated on NIX bind to LC3, and then bind to the GABAR complex to form the LC3-GABARAP complex, which targets LC3 to damaged mitochondrial outer membrane, and eventually initiates mitophagy. In addition, BNIP3 plays a role in the control of ubiquitin-dependent mitophagy by initiating mitochondrial movement of Parkin and facilitating Parkin-mediated mitophagy (process as in Figure 3). Finally, BNIP3 inhibits the Rheb-mTOR pathway, which also leads to autophagy activation. Abbreviations: BNIP3, BCL2-interacting protein 3; NIX, NIP3-like protein X; ROS, reactive oxygen species; HIF-1, Hypoxia-inducible factor-1; LC3, Microtubule-associated protein 1 light chain 3; LIR, LC3-interacting region; GABARAP, GABA type A receptor-associated protein; Rheb, Ras homologue enriched in brain; mTOR, target of the rapamycin machinery.

Mitochondrial autophagy can also be activated via the BCL2-interacting protein 3 and NIP3-like protein X (BNIP3/NIX) pathway. BNIP3 and NIX, situated in the mitochondrial outer membrane, are hypoxia-inducible factors and are characterized by low expression in physiological states. The latest studies have revealed the role of BNIP3 in controlling apoptosis, alongside its part in mitochondrial quality management through mitophagy (Wang et al., 2013; Lampert et al., 2019). Thomas et al. (2011) reported that, unlike PINK1 and Parkin, BNIP3 and NIX bind directly to autophagosomes, contributing to the activation of mitophagy. BNIP3 and NIX exhibit a 56% protein sequence similarity, and both contain BCL2 homology domain-3 (BH3). Under normal conditions, the interaction between BCL-xl/BCL2 and the BH3 of Beclin-1 results in the formation of Beclin-1/BCL-xl and Beclin-1/BCL2 complexes, effectively inhibiting autophagy. In response to stress, BNIP3/NIX are activated by hypoxia-inducible factor-1 (HIF-1) and interact with BCL-xl/BCL2 to release Beclin-1, subsequently activating mitophagy (Choubey et al., 2021). Furthermore, the control of BNIP3/NIX-mediated mitophagy involves phosphorylation—when BNIP3 Ser17 and Ser24 residues are phosphorylated, BNIP3 attaches to Microtubule-associated protein 1 light chain 3 (LC3) or to LC3 homolog GABA type A receptor-associated protein (GABARAP) through the LIR motif. Nevertheless, when NIX Ser34 and Ser35 residues undergo phosphorylation, NIX forms a bond with LC3, leading to the subsequent binding of LC3 to the γ-aminobutyric acid receptor-associated protein (GABAR) complex, ultimately resulting in the formation of the LC3-GABARAP complex. This complex targets LC3 in the damaged mitochondrial outer membrane and ultimately triggers mitophagy (Zhu et al., 2013; Rogov et al., 2017). BNIP3 contributes to ubiquitin-dependent mitophagy by inducing the mitochondrial movement of Parkin and promoting Parkin-mediated mitophagy. Additionally, BNIP3 inhibits the Ras homolog enriched in the brain (Rheb)/mTOR pathway and initiates autophagy (Figure 4) (Lin et al., 2014). Recent studies demonstrated that RPE in AMD has reduced levels of PINK1 and Parkin, and BNIP3/NIX-mediated mitophagy is the key factor in preserving mitochondrial equilibrium (Fisher et al., 2022; Jiménez-Loygorri et al., 2023). Esteban-Martínez and Boya (2018) reported that in response to metabolic stress, the destabilization of HIF1A/HIF-1 led to the upregulation of mitophagy receptor BNIP3/NIX. Furthermore, BNIP3L-dependent mitophagy-induced metabolic shifts in glycolysis are required for retinal ganglion cell (RGC) neurogenesis and the regulation of pro-inflammatory/M1-type macrophage polarization, which is vital for degenerative diseases such as AMD.

### 2.3 Mitophagy mediated by FUNDC1

Mitophagy can also be activated via the FUN14 domain-containing 1 (FUNDC1) pathway. FUNDC1 is a protein located in the outer membrane of the mitochondria, and its increased expression has been reported to trigger mitophagy (Kuang et al., 2016; Wu et al., 2016). The identification of FUNDC1 as a mitophagy receptor has been confirmed by recent studies (Mao et al., 2022). Wu et al. (2016) suggested that the site of FUNDC1 action is the mitochondria-associated endoplasmic reticulum membrane (MAM). FUNDC1, as a MAM-associated protein (Li et al., 2014), has an LIR motif at the amino-terminus that engages with LC3, and the deletion or structural changes to the LIR motif impede the connection between FUNDC1 and LC3, resulting in the downregulation of mitosis. The phosphorylation of FUNDC1, similar to BNIP3/NIX and BNIP3, can have either a positive or negative effect. Under physiological conditions, Src and casein kinase 2 prevent FUNDC1 from binding to LC3 by phosphorylating the Tyr18 residue of LIR, reducing mitophagic activity. When hypoxia or mitochondrial depolarization occurs, phosphoglycerate mutase family member 5 (PGAM5) induces the dephosphorylation of Ser13 (Liu et al., 2012; Chen et al., 2014); the dephosphorylated FUNDC1 then interacts with LC3 to activate mitophagy. Simultaneously, the deubiquitinating enzyme ubiquitin specific peptidase 19 (USP19), which is located in the endoplasmic reticulum, accumulates at the MAM and binds to the mitochondrial outer membrane protein FUNDC1, inducing its deubiquitination, promoting the oligomerization of dynamin-related protein 1 (DRP1), and resulting in mitochondrial fission (Zhang et al., 2022). Additionally, the unc-51-like autophagy-activating kinase 1 (ULK1) complex can translocate into the mitochondria, contributing to the phosphorylation of FUNDC1 Ser17 and the activation of mitophagy (Wu et al., 2014; Mercer et al., 2018). Another mitochondria-associated protein, nucleotide-binding oligomerization domain (NOD)-like receptor X1 (NLRX1), an immune system regulator, is known to diminish inflammatory responses, reduce ROS generation, and regulate autophagy. Increased NLRX1 expression reduces the relative levels of FUNDC1 phosphorylation and NLRP3 inflammasome-associated proteins in ARPE-19 cells, thereby preventing the development of AMD (Wang et al., 2023a). The exact mechanism of FUNDC1 action in mitophagy in AMD is not well characterized; however, studies suggest that the FUNDC1 pathway plays an integral role in obesity and metabolic disorders (Tan et al., 2018; Ren et al., 2020), as the excessive intake of saturated fatty acids affects the stability of mitophagy receptor FUNDC1 and mitochondrial mass, leading to mitochondrial dysfunction, obesity, and metabolic disorders (Chen et al., 2023). Wu et al. (2019) also reported that FUNDC1 deficiency in mice inhibits mitophagy, while a high-fat diet impairs mitochondrial function, resulting in high oxidative stress and heightened inflammatory responses. According to these results, the mitophagy receptor FUNDC1 is essential for maintaining mitochondrial quality, controlling inflammatory responses, and regulating metabolic disorders, possibly via mitogen-activated protein kinase (MAPK) signaling.

### 2.4 Mitophagy mediated by AMPK

**FIGURE 5:**
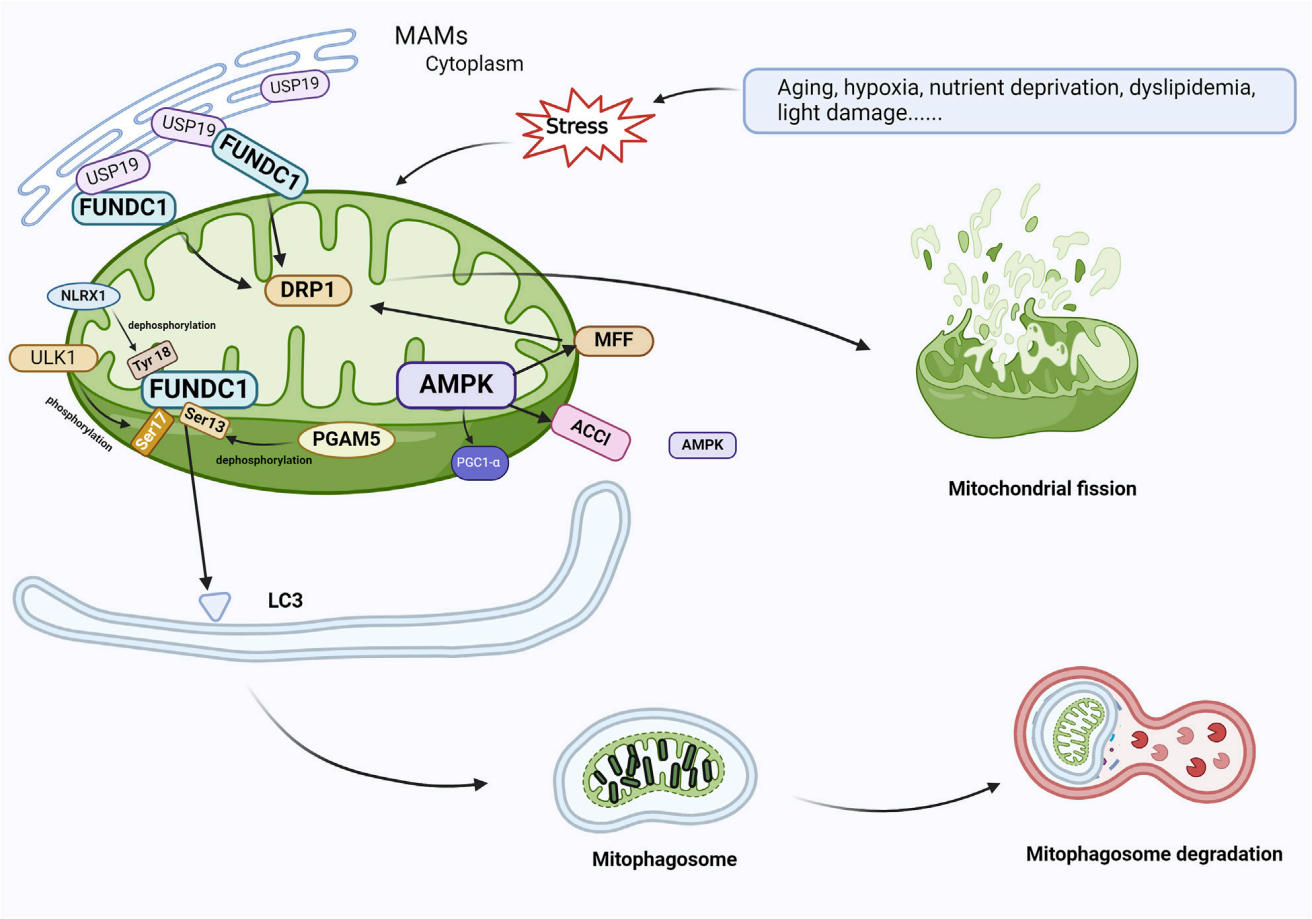
FUNDC1 and AMPK are involved in the regulation of mitophagy. In response to cellular stress, PGAM5 induces the dephosphorylation of Ser13, and FUNDC1 and LC3 interact to induce mitophagy. At the same time, USP19, which accumulates on MAM, attaches to FUNDC1 and triggers deubiquitination of FUNDC1, which promotes oligomerization of DRP1 and leads to mitochondrial fission. ULK1 complex translocates to mitochondria and phosphorylates Ser17 of FUNDC1 to initiate mitophagy, and NLRX1 activates FUNDC1 by dephosphorylating FUNDC1 Tyr 18 and induces mitophagy. During AMPK-mediated mitophagy, ACCl and MFF on the mitochondrial outer membrane, which are substrates for the action of AMPK, contact AMPK in the cytoplasm, resulting in the localization of AMPK within or near mitochondria. AMPK phosphorylates MFF and binds to DRP1 in response to oxidative stress, leading to mitochondrial fission, which initiates mitophagy and elimination of damaged mitochondria. Abbreviations: FUNDC1, FUN14 domain-containing 1; AMPK, AMP-activated protein kinase; PGAM5, phosphoglycerate mutase family member 5; USP19, Ubiquitin Specific Peptidase 19; MAM, mitochondria-associated endoplasmic reticulum membrane; DRP1, dynamin-associated protein 1; ULK1, Unc-51-like autophagy-activated kinase 1; NLRX1, nucleotide-binding oligomerization domain (NOD)-like receptor X1; ACC1, acetyl coenzyme A carboxylase 1; MFF, mitochondrial fission factor.

AMP-activated protein kinase (AMPK) is also related to the regulation of mitophagy. AMPK, a ubiquitously expressed serine/threonine kinase, is a highly conserved sensor of cellular energy and nutritional status and a major regulator of cellular metabolism (Rey and Tamargo-Gómez, 2023). AMPK activation plays a role in controlling oxidative stress, inflammation, glycolipid metabolism, mitophagy, and other functions (Cai et al., 2023; Feng et al., 2023; Guo et al., 2023; Zhong et al., 2023; Zhu et al., 2023). AMPK phosphorylates acetyl-CoA carboxylase 1 (ACC1) and mitochondrial fission factor (MFF) on the mitochondrial outer membrane. The interaction among phosphorylated ACC1, MFF, and AMPK in the cytoplasm facilitates the presence of AMPK in or around the mitochondria (Zong et al., 2019). DRP1 is the enzyme that catalyzes mitochondrial fission, while MFF is the primary receptor for DRP1 on the mitochondrial outer membrane. AMPK causes mitochondrial fragmentation by phosphorylating MFF in response to oxidative stress, initiating mitophagy and clearing damaged mitochondrial fragments (Toyama et al., 2016). In another model system, iron overload-induced mesenchymal stem cell (MSC) damage through the AMPK/MFF/DRP1 pathway increased MSC apoptosis, leading to mitochondrial fragmentation and enhanced autophagy (Zheng et al., 2018b). Furthermore, AMPK promotes mitophagy by directly phosphorylating PGC1-α (Figure 5) (Cantó et al., 2009).

Zhuang et al. (2022) reported that inhibition of the AMPK pathway induced RPE cell apoptosis, resulting in mitochondrial injury and the suppression of mitophagy. Xu et al. (2018) showed that increasing AMPK activity diminished DNA injury, reduced oxidative stress, and increased the production of mitochondrial energy, thereby protecting photoreceptors and RPE from acute damage, preventing their degeneration, and hindering the development of AMD. These findings confirm that AMPK-mediated mitophagy removes damaged mitochondria and protects mitochondrial function in response to oxidative stress. Furthermore, Salminen et al. (2012) reported that AMPK induced mitochondria generation by upregulating the transcription of PGC-1α target genes and removed impaired mitochondria via ULK1-dependent mitophagy. AMPK agonists also protect the cells by removing misfolded or damaged proteins from RPE cells through mitophagy.

Su et al. (2020) found that TXNIP was induced significantly in human retinal pigment epithelium, Muller glia, and cone photoreceptor cells under high-glucose conditions for five consecutive days, resulting in oxidative stress, ATP reduction, and decreased mitophagic flux. They proposed that TXNIP regulates Parkin/PINK1-mediated mitophagy in dopaminergic neurons under hyperglycemic conditions (Su et al., 2020). In addition, Devi observed that high glucose increased the expression of TXNIP at the mRNA and protein levels significantly in human RPE cell lines and primary human RPE cells. The upregulation of TXNIP was associated with mitochondrial membrane depolarization, fragmentation, and mitophagy access to lysosomes (Devi et al., 2019). Furthermore, Singh proposed that TXNIP leads to mitochondrial dysfunction, oxidative stress, dysfunctional mitophagy phagocytosis, lysosomal instability and inflammation in DR (Singh et al., 2018). The Nrf2/ARE signaling pathway plays an important role in the coordination of mitochondrial biogenesis and mitophagy, and it is involved in regulation of the expression of protective genes against oxidative stress, and regulation of mitochondrial biology and mitophagy. Consequently, it may be an important target for drugs for the treatment of neurodegeneration (Gureev et al., 2020). All the findings above suggest that TXNIP-mitochondria-lysosome and Nrf2-P62 also mediate mitophagy.

## 3 Mitophagy: a promising therapeutic target for AMD

As summarized in the previous section, mitophagy is strongly associated with the pathogenesis of AMD. Therefore, drugs that activate mitophagy in RPE cells could provide a novel therapeutic strategy for AMD. Recently, numerous mitophagy modulators that either activate or inhibit mitophagy, including AICAR (5-aminoimidazole-4-carboxamide ribonucleotide), an AMP analogue that maintains the optimal function of mitochondria (Ebeling et al., 2022), PGC-1α (an important regulator of mitochondrial biosynthesis) (Hyttinen et al., 2021), human retinal progenitor cells (hRPCs) (Yu et al., 2021), the mitochondria-targeted antioxidant triphenylphosphine (TPP)-nicotinic acid (Kim et al., 2021), melatonin (Mehrzadi et al., 2020), the mitochondrial activator PU-91 (Nashine et al., 2019), the mitochondria-derived peptide variant Humanin G (HNG) (Nashine et al., 2017), and the mitochondria-targeted antioxidant SkQ1 (Telegina et al., 2020; Fisher et al., 2022), have been discovered. mtDNA repair could be another target for improving mitochondrial function. Poly (ADP-ribose) polymerase (PARP1) active base excision repair (BER) and microhomology-mediated end-joining (MMEJ), among others, restore single- and double-strand breaks in mtDNA. Similar to nuclear DNA, nucleic acid complexes of multiple proteins can protect mtDNA from histone binding-induced ROS damage. However, owing to the complexity of mtDNA, its repair needs to be further investigated (Kaarniranta et al., 2020). Furthermore, these studies are still limited to animal and preclinical experiments, and the potential side effects during AMD treatment must be evaluated. A recent study demonstrated that augmenting mitophagic activity in individuals with AMD can impede the progression of the disease (Amini et al., 2023). The complexity of AMD pathogenesis should not be overlooked, as it encompasses a multitude of signaling crosstalks connecting the various layers of retinal structures to the choroid; as a result, positive outcomes are often difficult to achieve using single-agent therapy.

## 4 Role of TCM in mitophagy regulation in AMD

TCM treats diseases through various active ingredients, which possess antioxidant, anti-ageing, immunomodulatory, and anti-inflammatory properties as well as the ability to regulate mitophagy, and could thus be considered as an alternative therapeutic strategy for AMD. In China, *Astragalus mongholicus* Bunge, *Poria cocos* (Schw.) Wolf, *Plantago asiatica* L., *Atractylodes macrocephala* Koidz., *Angelica sinensis* (Oliv.) Diels, *Panax ginseng* C.A.Mey., *Salvia miltiorrhiza* Bunge, *Curcuma longa* L. (Pfahler et al., 2022; Vallée, 2022), *Rehmannia glutinosa* (Gaertn.) DC., *Lycium chinense* Mill., and *Glycyrrhiza glabra* L. (Kozlowski et al., 2015; Lee et al., 2015; Peng et al., 2016; Chen et al., 2021; Li et al., 2022a; Cho et al., 2023; Wei and Tong, 2023) are commonly used for the treatment of AMD. Studies have demonstrated that *Fructus lycii* can selectively activate and regulate the AMPK and VEGF pathways to enhance mitophagy, thereby preventing the development of retinopathy (Yang et al., 2022c). Another TCM, Mingmu Di Huang Pill, treats AMD by activating the expression of autophagy adaptor-SQSTM1 and AMPK phosphorylation, which can promote the autophagic degradation of Kelch-like ECH-related protein 1 (Keap1), safeguarding RPE cells against oxidative stress damage (Chen et al., 2022) (the main active ingredients, pharmacological actions, and mechanisms of these Chinese medicines are summarized in Table 1). Furthermore, various natural products from TCM, including berberine (BBR), curcumin, artemisinin, paeoniflorin, quercetin, luteolin, naringenin, ivytin (Cao et al., 2022b), urinary apolipid A (UA), ferulic acid, and astaxanthin (Lewis Luján et al., 2022), have been reported to have a mitophagy-regulating effect in numerous diseases by activating several specific pathways, including AMPK, PINK1/Parkin, BNIP3, FUNDC1, as well as LC3 proteins, followed by the induction of autolysosome formation. For example, UA, a novel mitophagy enhancer, initiates mitophagy by decreasing MMP without disrupting the mitochondrial respiratory chain and ROS production. Furthermore, it activates mitophagy by inducing the proper mitochondria function in a dose-dependent manner (Lu et al., 2023) and improves immune function through the PINK1/Parkin-mediated pathway (Denk et al., 2022). A previous study proposed that BBR has anti-inflammatory, antioxidant, antimicrobial, hypotensive, and gastric mucosa protective properties (Wang et al., 2017) as well as the ability to prevent oxidative damage triggered by hydrogen peroxide in human RPE cell line D407 via the activation of AMPK, indicating the potential therapeutic application of BBR for AMD (Li et al., 2018). Wang et al. (2023b) also demonstrated that BBR could act as a potential inducer of mitophagy, inhibiting PINK1 promoter methylation, reversing D-ribose-induced mitochondrial dysfunction, and restoring mitophagy via the PINK1/Parkin pathway, thereby attenuating the ageing process. Lu et al. (2023) reported that curcumin, a diketone extracted from the rhizomes of plants in the Zingiberaceae and Araceae, could potentially enhance Parkin-dependent mitophagy through the AMPK/transcription factor EB (TFEB) signaling pathway to diminish oxidative stress-induced injury to the intestinal barrier and mitochondrial dysfunction (Zhang et al., 2023). By activating AMPK, paeoniflorin, a monoterpene glycoside derived from *Paeonia lactiflora*, mitigates oxidative stress associated with Nox1/ROS in RPE cells, mitochondrial damage, and endoplasmic reticulum stress while protecting ARPE-19 cells and arresting the advancement of retinal degenerative disorders like AMD (Zhu et al., 2018) (Table 2). The latest evidence suggests that TCM or its active components could regulate mitophagy-related proteins, presenting a promising approach to treating AMD.

**TABLE 1:** Main active ingredients, pharmacological actions, and mechanisms of traditional Chinese medicine herbs and botanical drug decoction frequently used to treat AMD.

Single herb	Active ingredients	Pharmacological actions	Mechanisms	References
Poria	Poricoic acid A	Anti-inflammatory, antioxidant, immunomodulatory	Induction of mitochondrial dysfunction, endoplasmic reticulum stress and activation of the AMPK pathway	Yang et al. (2022a)
		Hypoglycemic and antifibrotic	Induction of mitophagy by downregulation of FUNDC1	Wu et al. (2023)
Radix *Angelicae sinensis*	*Angelica sinensis* polysaccharide	Antioxidant and anti-apoptotic	Downregulation of BNIP3 with the activation of mTOR and Notch signaling pathways	Xue et al. (2019)
Radix Panax Ginseng	Ginsenoside Rg1	Attenuates apoptotic and fibrotic responses	Enhancement of SIRT1/PINK1/Parkin-mediated mitophagy	Guan et al. (2023)
		Antioxidant stress	Increased expression of PINK1 and p-AMPK	Xu et al. (2019)
Radix Astragali	Astragaloside IV	Anti-ageing	Activation of Parkin-mediated mitophagy	Li et al. (2022b)
		Antioxidant stress	Enhancement of and restoration of mitochondrial function in an AMPK-dependent manner	Zhu et al. (2022a)
	Astragalus polysaccharide	Anti-apoptotic	Increased expression of BNIP3	Zhang et al. (2021)
Rhizoma *Atractylodis macrocephalae*	Atractylenolide III	Antifibrotic	Activation of AMPK signaling pathway	Huang et al. (2022)
		Antioxidant, antitumor, antiallergic reaction, antimicrobial and cognitive protection	Activation of the AMPK/SIRT1 signaling pathway	Li et al. (2022c)
*Salvia miltiorrhiza*	Tanshinone IIA	Anti-inflammatory, antioxidant, and anti-apoptotic	Activation of the AMPK/mTOR autophagy pathway by enhancing AMPK phosphorylation and the expression of Bcl-2/Bax, Beclin-1, LC3II/I, and SOD2, while diminishing mTOR phosphorylation, and the expression of NOX2, cleaved caspase-3/caspase-3	Si et al. (2023)
		Antioxidant, anti-apoptotic, regulation of mitophagy	Inhibition of the AMPK/Skp2 pathway by attenuating Parkin-mediated mitophagy	He and Gu (2018)
	Sodium Tanshinone IIA Sulfonate	Antioxidant, anti-inflammatory, anti-apoptotic, regulation of mitochondrial homeostasis	Inhibition of the overproduction of mitochondrial ROS via the AMPK pathway	Zhu et al. (2022b)
*Curcuma longa*	Curcumin	Anti-inflammatory, antioxidant, maintenance of mitochondrial homeostasis	Promotion of AMPK/PINK1/Parkin-mediated mitophagy	Jin et al. (2022)
		Antioxidant, anti-ageing, autophagy activation	Regulation of the SIRT1/AMPK/mTOR pathway, induction of autophagy	Yang et al. (2022b)
Mingmu Di Huang Pill	Quercetin, lignans and naringenins, strychnosides, paeoniflorin, salvinorin, etc.	Antioxidant, anti-apoptotic	Activation of autophagy articulator-SQSTM1 by AMPK-mediated degradation of autophagic Kelch-like ECH-related protein 1 (Keap1)	Chen et al. (2022)

**TABLE 2:** Natural chemical components and Mitophagy.

Natural chemical components	Pharmacological actions	Mechanisms	References
Urinary apolipid A	Anti-aging, anti-inflammatory, immunomodulatory, anti-tumor	1) Triggering mitophagy by decreasing MMP without interrupting ROS production and the mitochondrial respiratory chain 2) Improvement of immunity through PINK1/Parkin-related pathway	Denk et al. (2022), Lu et al. (2023)
Berberine	Antioxidant, anti-inflammatory, anti-aging, antibacterial, hypotensive, gastric mucosa protection	1) Inhibition of H_2_O_2_-induced oxidative damage in human retinal pigment epithelial cell line D407 cells by activation of AMPK 2) Restoration of mitophagy via the PINK1-Parkin pathway, thereby attenuating the aging process	Wang et al. (2017), Li et al. (2018), Wang et al. (2023b)
Paeoniflorin	Anti-inflammatory, antioxidant, immunomodulatory	Alleviating Nox1/ROS-associated oxidative stress, mitochondrial dysfunction, and endoplasmic reticulum stress in retinal pigment epithelial cells through activation of AMPK.	Zhu et al. (2018)

## 5 Conclusion and future directions

Mitophagy is an important mitochondrial quality control mechanism that selectively eliminates dysfunctional mitochondria to maintain cellular homeostasis. The regulation of mitophagy is mediated mainly by PINK1/Parkin, BNIP3/NIX, FUNDC1, and AMPK, and its dysfunction is closely related to AMD development. The use of drugs to increase mitophagy in RPE cells provides new ideas for AMD treatment.

Exploration of the relationship between TCM and mitophagy could offer key insights. The findings of such studies could broaden the scope of research on TCM theories by providing insights into how TCM can prevent and control AMD. In recent years, very few reports on TCM treatment of AMD by modulating mitophagy-related pathways have been published. In the present review, the authors focused on the role of mitophagy-related pathways in AMD pathogenesis, drawing on previous studies. In addition, the authors demonstrated potential ways via which TCM could modulate mitophagy to improve AMD based on three aspects: single botanical drugs, botanical drug decoction, and natural chemical components of TCM. However, although multiple pathways can mediate mitophagy, the correlation between the mediating pathways and the exact mechanism of mitophagy-induced AMD has not been elucidated. In future, more animal experiments and clinical experiments should be conducted under various pathological mechanisms to explore the role of mitophagy in AMD. In addition, studies on the regulation of mitophagy by TCM considering specific pharmacodynamics, target pathway, and mechanism of action should be carried out, to clarify the pathways through which the different active ingredients in single botanical drugs regulate mitophagy, and the mitophagy pathways via which different dosages and combinations of TCM could influence the overall AMD regulation, with a view to provide novel clinical insights and methods for the treatment of AMD with TCM.
